# Supraphysiologic Testosterone Therapy in the Treatment of Prostate Cancer: Models, Mechanisms and Questions

**DOI:** 10.3390/cancers9120166

**Published:** 2017-12-06

**Authors:** Osama S. Mohammad, Michael D. Nyquist, Michael T. Schweizer, Stephen P. Balk, Eva Corey, Stephen Plymate, Peter S. Nelson, Elahe A. Mostaghel

**Affiliations:** 1 Fred Hutchinson Cancer Research Center, Seattle, WA 98109, USA; osamasaad89@live.com (O.S.M.); mnyquist@fhcrc.org (M.D.N.); scwheize@uw.edu (M.T.S.); pnelson@fhcrc.org (P.S.N.); 2 Faculty of Medicine, Benha University, Benha 13518, Egypt; 3 School of Medicine, University of Washington, Seattle, WA 98195, USA; splymate@uw.edu; 4 Beth Israel Deaconess Medical Center, Boston, MA 02215, USA; sbalk@bidmc.org; 5 Department of Urology, University of Washington, Seattle, WA 98195, USA; ecorey@u.washington.edu

**Keywords:** high dose testosterone, supraphysiologic androgen, bipolar androgen therapy, biphasic, BAT, castration resistant prostate cancer, CRPC

## Abstract

Since Huggins defined the androgen-sensitive nature of prostate cancer (PCa), suppression of systemic testosterone (T) has remained the most effective initial therapy for advanced disease although progression inevitably occurs. From the inception of clinical efforts to suppress androgen receptor (AR) signaling by reducing AR ligands, it was also recognized that administration of T in men with castration-resistant prostate cancer (CRPC) could result in substantial clinical responses. Data from preclinical models have reproducibly shown biphasic responses to T administration, with proliferation at low androgen concentrations and growth inhibition at supraphysiological T concentrations. Many questions regarding the biphasic response of PCa to androgen treatment remain, primarily regarding the mechanisms driving these responses and how best to exploit the biphasic phenomenon clinically. Here we review the preclinical and clinical data on high dose androgen growth repression and discuss cellular pathways and mechanisms likely to be involved in mediating this response. Although meaningful clinical responses have now been observed in men with PCa treated with high dose T, not all men respond, leading to questions regarding which tumor characteristics promote response or resistance, and highlighting the need for studies designed to determine the molecular mechanism(s) driving these responses and identify predictive biomarkers.

## 1. Introduction

Since the landmark studies of Huggins and colleagues showed the androgen-sensitive nature of prostate cancer (PCa) [1], suppression of systemic testosterone (T) levels remains the most effective initial therapy for advanced disease. Although initially highly effective, standard androgen deprivation therapy (ADT) is characterized by the emergence of resistant tumors over a period of 18–20 months. Median survival after progression, termed castration resistant prostate cancer (CRPC), is between 1–2 years. An important aspect of CRPC is reactivation of androgen-receptor (AR) signaling, as demonstrated by analyses of metastatic tumors showing that essentially all known androgen regulated genes are expressed, including putative drivers of carcinogenesis (e.g., Transmembrane Protease, Serine 2-ETS-related gene (*TMPRSS2-ERG)* rearrangements).

From the inception of clinical efforts to suppress AR signaling by reducing AR ligands, it was also recognized that the administration of T to men with CRPC can result in substantial clinical responses, though the reports were largely anecdotal and remissions were highly variable, potentially due to variability and inadequate increases in the levels of circulating T that were achieved [2,3,4]. In contrast, abundant data from preclinical models have reproducibly shown biphasic responses of hormone-sensitive cancers, whereby at physiological T concentrations proliferation is induced, but at higher, supraphysiological T (SPT) concentrations, proliferation is suppressed and in some instances apoptotic programs are engaged [5,6,7,8]. Though often considered to be an in vitro phenomenon, recent proof of principle trials using SPT therapy—two in men with CRPC and one in hormone sensitive PCa produced promising results, showing radiographic response rates of ~50% in men with CRPC, and favorable prostate specific antigen (PSA) responses in those with hormone naïve PCa [9,10,11].

Notably, biphasic responses to hormone concentrations are not unique to PCa and the AR: When exposed to estradiol (E2), estrogen receptor (ER)-responsive MCF-7 breast cancer cell line adapted to proliferate in the absence of estradiol undergo an apoptotic response [12,13,14]. E2 also has a biphasic effect on the growth of the rat pituitary line GH3 [6]. To date, there is a lack of unifying mechanisms to explain these effects, though cellular pathways, particularly involving cell cycle control, provide insights in some systems.

Many questions regarding the biphasic responses of prostate tumors to supraphysiologic androgen concentrations remain, primarily regarding the mechanisms driving this response and how best to exploit this phenomenon clinically. To date, efforts to enhance efficacy of SPT have focused on concomitant manipulation of the androgen receptor (AR), i.e., androgen cycling to induce AR upregulation and increased sensitivity, but preclinical studies suggest other approaches and drug combinations may be reasonable to pursue. Here we review the preclinical and clinical literature on androgen-mediated growth repression and discuss cellular pathways and mechanisms likely to be involved in mediating this response. Although meaningful clinical responses have now been observed in men with CRPC treated with SPT [9], not all men respond, leading to questions regarding which tumor characteristics promote response or resistance, highlighting the need for studies designed to determine the molecular mechanism(s) driving these responses and identify predictive biomarkers.

## 2. Physiologic Role of AR in Growth Repression

The AR plays a critical role in the normal development of the prostate gland, although initial morphogenic activity occurs via mesenchymal AR, not epithelial AR [15]. In the mature prostate, the small fraction of epithelial cells that are proliferating are localized to the basal compartment, and do not express AR protein, whereas luminal secretory cells which express AR are quiescent. Introducing the AR into benign prostate epithelial cells (PrEC), or activating AR function, results in cell growth arrest and subsequent differentiation toward a luminal phenotype. Detailed studies conducted by Isaacs et al. determined that the induction of AR activity in PrEC resulted in irreversible growth arrest in G0, with the maintenance of viability, metabolism, and the expression of proteins that are associated with terminally-differentiated prostate epithelium, such as PSA [16,17]. Studies using genetically-engineered mouse models (GEMs) showed that eliminating AR in prostate epithelium results in a hyper-proliferative cell state with loss of cell differentiation [18,19,20]. These and other studies provide compelling evidence that in benign cells with intact mechanisms for regulating cell proliferation, the AR functions to promote terminal differentiation and a quiescent, G0 state.

## 3. Oncogenic Role of AR in Prostate Cancer Progression

In contrast to its role in promoting differentiation in normal prostate epithelial cells, AR signaling in PCa acquires a critical oncogenic role and promotes growth and survival. The mechanism for this conversion is not fully understood but appears to involve in part a gain of function in AR induced regulation of myelocytomatosis oncogene cellular homolog (MYC) expression [16,17]. During progression to castration resistance, PCa acquire further changes directed at maintaining AR signaling in a low androgen environment. These changes include AR overexpression, AR mutations that broaden ligand specificity and/or confer sensitivity to adrenal androgens, induction of constitutively active truncated AR splice variants, alterations in AR coactivators and/or corepressors that modulate AR stability and ligand sensitivity, and activation of the AR or downstream regulatory molecules by “cross talk” with other signaling pathways [21,22,23,24,25,26,27,28,29,30,31,32,33,34,35,36,37,38,39,40,41,42,43]. Restoration of full length AR expression and signaling in a xenograft model was both necessary and sufficient to drive progression from androgen-dependent to castration resistant growth, sensitizing cells to proliferate in 80% lower androgen concentrations [33]. Importantly, in these models ligand binding was required for castration resistant growth, and modest increases in AR expression were sufficient to support signaling in a low androgen environment.

The clinical relevance of continued AR signaling in promoting CRPC tumor growth is confirmed by the clinical responses to agents targeting residual androgen pathway activity including the striking clinical response observed with novel ligand synthesis inhibitors such as abiraterone (ABI), and potent AR inhibitors such as enzalutamide (ENZ) [44,45,46,47,48,49,50].

## 4. Historical Observations on Androgen Therapy of Prostate Cancer

Interestingly, despite his clear demonstration that androgens were a critical driver of PCa progression, Huggins himself proposed that both hormonal deprivation and hormonal excess (which he termed hormonal interference) might be used for therapeutic benefit [51]. A number of case reports and small series were published between 1950 and 1980. While demonstrating some evidence of clinical benefit, these studies also showed adverse effects of T administration, though the doses were generally low, ranging from 25 mg–100 mg daily [2,52]. In a series of three patients, two experienced temporary symptomatic benefit. In a series of 10 men with CRPC treated with 100 mg T propionate 3 times weekly, one individual, near death at the time of treatment initiation, experienced an objective response lasting approximately 1 year, although five patients had subjective and objective evidence of deterioration including pain and pathologic fracture [3]. Two case reports also detail responses to T therapy, one patient with progression despite orchiectomy and hypophysectomy who responded to T with a decrease in serum acid phosphatase and symptomatic improvement, and more recent patient with CRPC treated with T gel replacement with a sustained PSA response lasting for nearly a year [53,54].

Importantly, simply discontinuing ADT and allowing androgen recovery to a eugonadal state does not appear to enhance survival; studies of intermittent androgen suppression in metastatic disease, which also allows gradual T recovery to physiological levels, demonstrated a trend toward inferior survival compared to continuous ADT [55]. In this regard castration resistant VCaP cells treated with sequentially higher T doses had less significant apoptotic responses to androgen withdrawal than those seen in VCaP cells exposed to a single high T “boost” [56].

## 5. Preclinical Observations on Androgen-Mediated Growth Repression of Prostate Cancer

Although historical support for the clinical benefit of androgen therapy for PCa has been mixed, the paradoxical inhibitory response of PCa to supraphysiological androgens has been demonstrated in multiple in vitro and in vivo studies. These studies are summarized below, and a more detailed review of findings in each pre-clinical model is provided in Appendix A. The LNCaP cell line is widely used as a model for mechanistic studies of PCa molecular biology, including AR function. Several groups have reproducibly demonstrated a biphasic proliferative response to androgen, with minimal proliferation in the absence of any androgen, high rates of proliferation at concentrations of dihydrotestosterone (DHT) or the non-metabolizable androgen (R1881) of 0.1 nM, and cell cycle arrest with concentrations of DHT/R1881 exceeding 1.0 nM (equivalent to ~5–10 nM T) [7,8,57]. This effect has been observed in other PCa lines that natively express the AR as well as cells engineered to express the AR (summarized in Appendix Table A1).

Within a tumor model, the response of cells to androgen-repressed growth is often more pronounced in castration resistant (CR) variants that have been derived after serial passage in androgen-depleted media, whereby the growth repressing effects can occur at 10–100 fold *less* androgen. Progression to the CR state is often accompanied by an increase in AR levels and transcriptional activity, suggesting the elevated AR levels in the more refractory tumor cells sensitizes these models to repression at lower androgen doses. In the transition from the androgen sensitive (AS) LNCaP 104S variant to the androgen independent (AI) 104R line (after 8–11 months in charcoal stripped (CS) media), the repressive dose of R1881 is left-shifted two orders of magnitude, from 1 nM (10^−9^) to 0.01 nM (10^−11^), accompanied by increased AR expression and activity suggesting an increased sensitivity to androgen signaling [58]. An AI subline of MDA PCa 2ba shifts from being androgen-stimulated for growth to being androgen suppressed for growth with an increase in AR levels [59,60]. Similar to LNCaP, the growth of CWR22 cells is biphasic and the suppression is left shifted in the AI line CWR22R [61].

Exemplifying the known heterogeneity of advanced disease, PCa cell lines demonstrate a diversity of androgen-mediated growth responses. Importantly, an androgen-repressed growth phenotype is not exclusively associated with elevated AR levels, nor do low AR levels preclude androgen-repressed growth. Some PC cells with relatively low AR such as ARCaP still show a strong androgen-repressed phenotype [62]. In contradistinction to CWR22 cells, 22Rv1, another AI line derived from CWR22R, is AS for growth without a biphasic response [63]. The mechanism(s) underlying these responses and why the growth repressive effect of SPT is not uniformly observed in all AR + PC cells are not understood. In tumors where the repressive response is left shifted with increased AR it is not known whether additional mechanisms are operative. Nor is it known to what extent the mechanism of AR-mediated growth-suppression occurs by similar or different mechanisms in the different androgen-repressed cell lines.

Notably, despite compelling preclinical evidence that the androgen-repressive effect is often magnified in CR tumor variants, clinical observations do not necessarily support this. In the early studies of Fowler and Whitmore 45 of 52 men had unfavorable responses to exogenous T, and the proportion of men who had an unfavorable response was higher in those who had been on prolonged hormone suppression (94%) compared to castration-naïve men (25%), or men in early stages of hormone suppression (36%) [64]. It is possible that while repressive responses to SPT may be enhanced in CR tumors with upregulated AR, growth-promoting responses to physiologic T may be similarly enhanced in CR tumors.

## 6. Contemporary Clinical Studies of Testosterone Therapy for Prostate Cancer

Several contemporary trials of T treatment have been conducted (summarized in Table 1), two in which physiologic T levels were achieved and minimal responses were observed [65,66], and three in which T levels achieved were truly supraphysiologic and a clear subset of men showed clinical responses [9,10,11].

### 6.1. Studies of Continuous Testosterone Treatment

Morris et al. conducted a Phase 1 trial of 12 men with CRPC who were treated with T via 5 mg transdermal patch or 1% gel for 1 week, 1 month, or until disease progression. T treatment resulted in raising T levels to the normal physiological range although DHT levels were supraphysiologic in a subset of men. One patient achieved a PSA decline of >50% from baseline, but no objective responses were seen, with a median time to progression of 84 days (range 23–247 days) [65].

Szmulewitz et al. conducted a randomized trial evaluating transdermal T at 25, 5.0 or 75 mg/day in 15 men with CRPC but minimal metastatic disease [66]. Notably, serum T levels increased from castrate to ~300 ng/dL, essentially a eugonadal concentration. Increases in PSA, which may rise with T replacement, rather than other objective measures of disease progression were responsible for the majority of progression events. (20%) of patients demonstrated a decrease in PSA (largest was 43%), with a median time to progression of 9 weeks (range: 2–96 weeks). One patient experienced symptomatic progression. There was no significant improvement in quality of life (QoL).

### 6.2. Studies of Bipolar Androgen Therapy (BAT)

In contrast to these studies in which T was administered in continuous manner, the group of Denmeade and Isaacs has pioneered an approach termed Bipolar Androgen Therapy (BAT) [67]. Overexpression of AR is one of the most common molecular hallmarks of CRPC [68], and it was hypothesized that by rapidly cycling T levels between the supraphysiological (~1500 ng/dL) and near-castrate (~150 ng/dL) range, adaptive changes in AR expression would be blunted, thereby delaying the emergence of resistance. Data from these investigators has also suggested that AR becomes critically involved in the DNA replication licensing required for PCa cell proliferation. As discussed more fully below, increased ligand may over-stabilize AR on DNA, preventing its degradation and inhibits DNA relicensing, resulting in cell death in the subsequent cycle [69,70,71].

Schweizer et al. reported the first clinical experience with this approach: 16 men with asymptomatic metastatic CRPC were treated with 400 mg of T intramuscularly (IM) monthly [9]. Notably, 2 days after T administration, serum T levels exceeded 1500 ng/dL (~50 nM) and fell to <200 ng/dL at the end of each 28 day cycle. PSA declines (≥50%) were observed in nearly one-third of patients, radiographic responses were observed in 50% of men, and four continued on treatment for ≥1 year. At progression, ADT or AR inhibitory therapy produced responses in 100% of men, suggesting that BAT may restore sensitivity to ADT [9]. Importantly, no patient developed worsening pain due to PCa, nor were there any other skeletal events or evidence of worsening urinary obstruction.

In a follow up study recently reported by Teply et al, men with CRPC who had progressed on ENZ went on to receive BAT (*n* = 30) [10]. This study documented similar activity in response to BAT, with 9 of 30 (30%) men achieving a ≥50% decline in PSA from baseline and 50% of patients achieving an objective radiographic response. Twenty-nine patients progressed on BAT and went on to be re-challenged with ENZ. Fifteen of 29 (52%) had a PSA decline ≥50%, however, there were no objective radiographic responses and time to progression was generally short following ENZ re-challenge.

In another study in hormone naive patients, 29 men with low metastatic burden or biochemically recurrent disease who achieved PSA < 4 ng/dL after 6 months of ADT were treated with alternating 3 month cycles of BAT (given as IM injections of 400 mg T cypionate or enanthate on days 1, 29 or 57), followed by 3 months of ADT alone [10]. The primary endpoint was the percent of patients with a PSA < 4 ng/dL after two rounds of BAT-ADT (i.e., following the 18-month treatment period). Serum androgen levels were not reported. Three of 29 patients were taken off study prior to completing two cycles due to concerns for early progression. However, the 26 patients that completed the study as designed achieved a PSA below their pre-treatment baseline, with 17/29 (59%) achieving the primary endpoint of PSA < 4 ng/dL after 18 months, including three patients who had an undetectable PSA (<0.2 ng/mL) at the18-month time point. Of 10 men with measurable disease, four complete and four partial responses were observed. Notably, five of seven patients who did progress to CRPC by the end of the study responded to subsequent treatment with anti-androgen, again suggesting that BAT may restore sensitivity to ADT Treatment was associated with favorable improvement in QoL, although QoL diminished over the course of each cycle of BAT, presumably due to T levels falling below the normal range.

BAT is currently being tested in a large (*n* = 180) randomized trial (NCT02286921; TRANSFORMER) in asymptomatic mCRPC patients who have failed on abiraterone. In this study, BAT is being compared with ENZ with a primary end point of progression-free survival (PFS). While the more substantive clinical benefit observed in the studies of BAT vs. other contemporary and historical studies of androgen treatment may reflect the bipolar dosing strategy, it is likely that it also is related to the fact that these are the only studies in which supraphysiologic androgens have been achieved. Importantly, the relative dearth of patients experiencing clinical deterioration on T treatment compared to historical studies highlights the importance of patient selection, which, in modern studies, was limited to asymptomatic patients with limited metastatic disease.

## 7. Proposed Mechanisms of Androgen-Mediated Growth Repression

**Figure 1:**
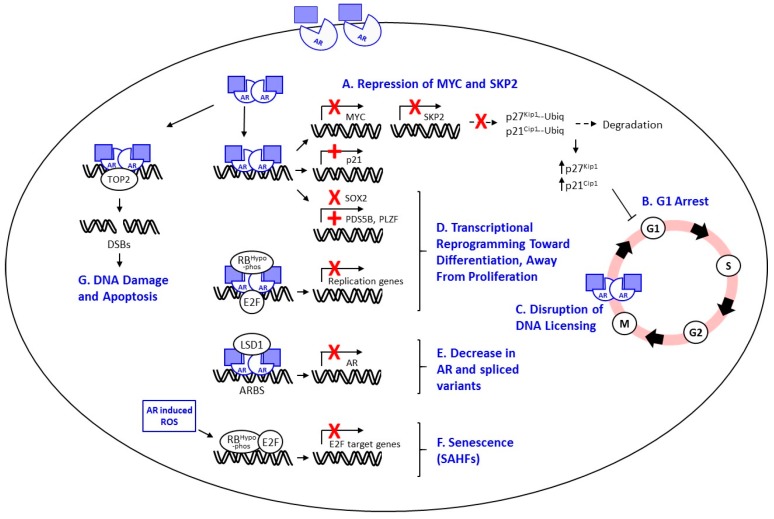
Potential mechanisms for repression of prostate cancer growth by high dose androgen. (**A**) AR activation in context of high dose androgen (denoted by light blue squares) may lead to transcriptional repression of myelocytomatosis oncogene cellular homolog (MYC) and its target gene S-phase kinase-associated protein 2 (SKP2), with loss of ubiquitin-mediated degradation of the G1 cyclin dependent kinase (CDK) inhibitors p21cip1 and p27kip1, leading to (**B**) G1 arrest. (AR can also directly induce expression of p21cip1 via an androgen response element (ARE) in its proximal promoter). (**C**) Ligand-dependent stabilization of AR during mitosis may inhibit AR degradation in M phase, preventing relicensing for DNA replication during G1 resulting in S phase arrest. (**D**) Androgen induced repression of genes that promote epithelial to mesenchymal transition (EMT) such as (sex determining region Y)-box 2 (SOX2), and expression of genes important in normal differentiation such as sister chromatid cohesion protein cohesion associated factor B (PDS5B (also known as androgen-induced proliferation inhibitor (APRIN)) and promyelocytic leukemia zinc finger protein (PLZF), may promote a more differentiated less aggressive cell state. Through recruitment of hypo-phosphorylated retinoblastoma protein (RB) to shared AR/RB/E2F binding sites, agonist-liganded AR represses genes involved in DNA replication, potentially leading to transcriptional reprogramming toward a less proliferative state. (**E**) Activated AR may act as a transcriptional repressor at certain AR binding sites (ARBS) via recruitment of lysine-specific histone demethylase 1 (LSD1) and demethylation of activating histone marks, resulting in decreased expression of full length AR and downstream generation of spliced variants. (**F**) AR-induced production of reactive oxygen species (ROS) leading to decreased RB phosphorylation and repression of E2F target genes may result in formation of senescence-associated heterochromatic foci (SAHFs). (**G**) Androgen signaling leads to co-recruitment of AR and topoisomerase II beta (TOP2B) and TOP2B-mediated DNA double stranded breaks (DSBs) in regulatory regions of AR target genes, potentially leading to DNA damage and apoptosis, particularly in the setting of DNA damage repair (DDR) deficiency (such as mutations in ataxia-telangiectasia mutated gene (ATM) or breast cancer 2 (BRCA2)). X: inhibition of gene expression; +: induction of gene expression.

Numerous studies utilizing PCa models with endogenously expressed AR, as well as cell models with exogenously driven increases in AR, have shown that increased AR expression and/or ligand driven activation may result in growth inhibition, variably attributed to cell cycle arrest in G1/S or the subG0/G1 phase, and/or frank DNA fragmentation and apoptosis [18,36,70,72,73,74,75,76,77]. A number of mechanisms underlying the growth inhibitory effects of SPT have been proposed (summarized in Figure 1) including: I. Cell Cycle Arrest; II. Repression of MYC and S-phase kinase-associated protein 2 (SKP2); III. Apoptosis; IV. Disruption of AR-Mediated DNA Licensing; V. Transcriptional Repression of AR and AR Variants; VI. Transcriptional Reprogramming and Differentiation; and VII. Induction of Cellular Senescence or Quiescence; VIII. Induction of DNA Damage.

### 7.1. Cell Cycle Arrest

De Launoit et al. showed that at the maximal proliferative dose of DHT (0.1 nM), the fraction of androgen-sensitive LNCaP cells in G0–G1 phase significantly decreased (at 36 and 48 h), reflected by an increase in cells in the S and G2/M phases, whereas at growth inhibitory doses of DHT greater than 0.1 nM, an increase in the number of cells in G0–G1 phase was observed, with a significant decrease in cells in the S and G2/M phases [78,79]. G1 arrest was also shown in the androgen independent LNCaP 104-R1 and 104-R2 sub-lines treated with 10 nM R1881, with the maximum decline in the S phase fraction at 72 h after androgen treatment [57]. However, R1881 had no effect on cell cycle distribution in the related androgen-insensitive LNCaP R2-Ad subline [80]. Treatment with 1 nM R1881 yielded a cell cycle arrest in the androgen-repressed MOP, ME, and JAC LNCaP sublines, with an increase in the proportion of cells arrested it the G1 phase and a compensatory decrease in cells in the S and G2/M phases [76].

SPT in PC-3 cells with exogenous expression of AR has also shown a G0/G1 growth arrest. Litvinov et al. reported that overexpression of AR in PC-3 cells caused growth inhibition via G1 arrest, associated with increased expression of the cyclin dependent kinase (CDK) inhibitor p21 (WAF1/CIP1) (a known AR-regulated gene via an ARE in its proximal promoter [81]), as well as an increase in p27kip1 and suppression of p45/SKP2 (normally upregulated in G1 to target the CDK inhibitor p27kip1 for proteosomal degradation) [69,82]. These findings were confirmed by Kokontis et al., using PC3 cells re-expressing either the wild type AR (PC3-AR) or the mutant LNCaP AR (PC3 LNCaP-AR) [83].

### 7.2. Repression of SKP2 and MYC

Exposure of LNCaP and LNCaP-derived sublines to SPT has been shown to repress the expression of SKP2 [84]. SKP2 is a substrate-targeting subunit of the SCF E3 ubiquitin ligase complex which phosphorylates p21cip1 and p27kip1, targeting them for ubiquitination and degradation. p27kip1 is a cyclin-dependent kinase inhibitor which is encoded by *CDKN1B* gene [85]. The encoded protein was shown to inhibit the cyclin E/CDK2 complex in androgen-treated LNCaP cells [57,79]. LNCaP cells treated with SPT show elevated levels of p27kip1 protein, consistent with impairment in the degradation program following the reduction of SKP2 [57,79,80,86].

One proposed mechanism involves AR-mediated repression of MYC (transcription), with concomitant repression of MYC target genes, which include the ubiquitin ligase SKP2, and thereby upregulation of the G1 cyclin dependent kinase (CDK) inhibitor p27Kip1 which is regulated by SKP2-mediated degradation [16,80,83]. Although not shown in PCa, SKP2 is a direct transcriptional target of MYC in tumor cells including leukemia and neuroblastoma [87,88]. Conversely, SKP2 regulates MYC ubiquitination and stability and serves as a transcriptional coactivator for MYC [89].

In the androgen-sensitive LNCaP 104-S cells, R1881 increased levels of MYC and SKP2 expression and decreased levels of p27kip1 [80]. In contrast, R1881 decreased the levels of MYC and SKP2 and increased p27kip1 in the more strongly androgen-repressed LNCaP 104-R cell lines [80,83]. Moreover, overexpression of this protein along with MYC countered androgen-mediated growth suppression in castration resistant 104-R LNCaP cells [80,83]. In the androgen-insensitive R2Ad cell line, R1881 did not alter the levels of MYC, p27kip1, or SKP2 [90].

SKP2 is also a transcriptional target of E2F [91], and changes in its expression may reflect AR-mediated changes in RB induction of E2F. Jiang et al. demonstrated that SKP2 transcription is directly suppressed by an RB family member, the p107 pocket protein, following SPT treatment [82]. Repressing p107 partially blocked SKP2 repression following exposure to SPT, though whether SKP2 or p107 inhibition altered SPT growth arrest was not determined. Notably, SPT was shown to reduce p107 phosphorylation, though the AR-regulated phosphatase (potentially induced) or kinase (potentially repressed) responsible for this effect was not identified.

To date, these findings have not been validated across models that respond or resist SPT, and the mechanism(s) by which MYC or SKP2 are regulated by SPT have not been identified.

### 7.3. Apoptosis

While the role of androgen has been shown to be anti-apoptotic in androgen-dependent PCa cells [92], androgen may also induce apoptosis in castration resistant cell lines. Treatment of MOP cells, a castration resistant and androgen-repressed subline of LNCaP, with 100 nM R1881 resulted in an increase in the apoptotic index, with 37% of cells showing nuclear fragmentation and inter-nucleosomal DNA breaks after 6 days of treatment [75]. In the castration resistant 104-R1 LNCaP cell line, AR promoted B-cell lymphoma 2 (BCL2) associated x, apoptosis regulator (BAX)-mediated apoptosis, was involved in mitochondrial translocation of BAX, and addition of androgen potentiated AR-related BAX translocation and the induction of apoptosis [77]. BAX is a member of the BCL-2 family that when activated, initiates apoptosis [93]. Knocking down of AR by siRNA in LNCaP 104-R1 cells resulted in failure of BAX to induce apoptosis. Similarly, while UV induced apoptosis in 40% of the parental LNCaP cells, only 9% of the cells with AR knockdown underwent UV-induced apoptosis [77]. Moreover, addition of androgen potentiated BAX-mediated apoptosis, as was seen in 37% of cells treated with 1 nM R1881 compared to only 19% cell death induced by BAX alone. Androgen also induced apoptosis in PC-3 cells engineered to overexpress AR [73]. However, AR-dependent UV-induced apoptosis was also achieved in the same LNCaP cell line in an androgen independent manner, via AR transcriptional downregulation of p21cip1 expression [94]. p21cip1 is known to have anti-apoptotic and tumor promoting functions, and in the LNCaP 104R model AR was shown to prime cells for apoptosis via down-regulation of basal p21cip1 expression [94].

### 7.4. Disruption of AR-Mediated DNA Licensing

Normally, licensing factors are degraded in M phase or early G1 phase to allow for relicensing and re-initiation of DNA replication in the next cell cycle [95]. Litvinov et al. and Isaacs et al. have shown AR to interact with the pre-RC and DNA replication machinery in early G1 phase, suggesting it may act as a “licensing” factor for initiation of DNA replication in the subsequent S phase [69,96]. They suggest that excessive ligand-dependent stabilization of AR during mitosis, due to either increased AR or increased ligand, inhibits AR degradation in M phase. This results in a fraction of AR remaining bound to origin of replication sites, preventing relicensing during G1 and resulting in S phase arrest. As AR levels increase with ADT and decrease with normalization of androgen levels they have proposed that rapid androgen cycling between ADT and supraphysiogic androgen levels will prevent the adaptive down regulation of AR levels (that can occur in response to a slow rise in androgens) that would allow re-licensing and cause a poor inhibitory response to subsequent androgen treatment.

Using LNCaP, LAPC4 and CWR22Rv1 PCa cells, a cyclic proteasome-dependent degradation of AR during G1 was observed, along with co-immunoprecipitation (co-IP) of AR with replication complexes (RC), and co-IP of AR with origin of replication complex 2 (ORC2) in four of seven human CRPC metastases, suggesting AR may function as a licensing factor for DNA replication in cells that are androgen-sensitive for growth. In contrast, they do not observe binding of AR to RCs in cells in which liganded AR does not drive growth (the E006AA PCa line, and a prostate stromal line) [69,71]. The impact of high dose androgen on these parameters was not directly tested in LAPC4 or 22Rv1 cell lines. However, in parental LNCaP cells, a LNCaP derivative with castration resistant growth, in 22Rv1 cells overexpressing AR, and in PC-3 cells overexpressing AR, androgen-mediated growth repression was associated with a marked increase in the percent of mitotic cells with detectable AR expression (i.e., 85% vs. 0.5% in untreated), consistent with a role of AR degradation in permitting relicensing of DNA for subsequent replication. in these cells.

However, while cyclic degradation of AR in 22Rv1 cells, and association of AR with RC’s in 22Rv1 and VCaP cells was similarly observed, a repressive effect of androgen on 22RV1 does not occur; androgen remains stimulatory at 10^–6^ M T in the 22Rv1 model [63]. These observations suggest that a potential impact of ligand on AR stabilization and interference with proper DNA re-licensing may not be the mechanism by which SPT mediates growth inhibition in all cases.

### 7.5. Transcriptional Repression of AR and AR Variants

To the extent that AR and AR splice variants are drivers of CRPC progression, mechanisms that repress their expression may lead to decreased cell growth. Studies by the Balk group have shown that AR gene expression is directly repressed by the AR through recruitment of lysine-specific histone demethylase 1 (LSD1) to an AR Binding Site (ARBS) region termed ARBS2 of which a segment of ~400 bp is highly conserved across species [97]. Agonist liganded AR was shown to decreases AR gene expression in castration resistant VCS2 cells (derived from VCaP cells) by functioning as a transcriptional repressor through recruitment of LSD1 and demethylation of histone 3 lysine 4 (H3K4)me1,2 [97]. AR also repressed aldo-keto reductase family 1 member C3 (AKR1C3) and hydroxysteroid 17-beta dehydrogenase 6 (HSD17B6) through a similar LSD1 dependent mechanism, indicating that the agonist liganded AR directly mediates a physiological intracellular negative feedback loop to regulate AR activity. Moreover, in all cases the androgen-stimulated down-regulation was decreased or abrogated by treatment with the LSD1 inhibitor pargyline. The role of LSD1 inhibition in the context of SPT has not been explicitly evaluated, although loss of LSD1 or LSD1 activity has the potential to abrogate SPT effects by inhibiting generation of AR repressive complexes on key targets, or may enhance SPT effects by increasing levels of AR itself.

Whether SPT results in dynamic changes of AR expression associated with growth inhibition in the clinical setting has not been assessed, but has been suggested in several pre-clinical models in vivo. Thelen et al. compared the growth of castration sensitive VCaP cells maintained in 10% fetal bovine serum (FBS) to a subline adapted to growth in 1 nM T after implantation in intact (non-castrate) nude mice [56]. Notably, the VCaP cells grown in FBS showed a significant growth disadvantage compared to the subline adapted to T in cell culture. Transcript levels of AR and ARV7 were rapidly downregulated by 1 nM T in the VCaP cells maintained in 10% FBS in vitro, and were also lower in the xenografts grown in intact mice than in the parental cells passaged in 10% FBS.

Nakata examined growth inhibition by androgen in JDCaP-hr, an AR and ARV7 positive cell line derived from the castration recurrent outgrowth of a JDCaP xenograft (generated from the skin metastasis of a Japanese CRPC patient) [98,99]. Expression of full length AR (AR-FL) and splice variant 7 AR (AR-V7) mRNA was upregulated by 10-fold in JDCaP-hr compared with that in JDCaP. T suppressed the growth of JDCaP-hr in vitro and in vivo in association with downregulation of AR and ARV7 expression, while silencing of AR-V7 but not AR-FL markedly suppressed cell growth.

A recent trial of BAT in patients who progressed on ENZ did not show a clear correlation between response and modulation of AR-FL or AR-V7 transcript levels in circulating tumor cells [11]. However, further studies, potentially with assessment of tumor AR expression, are needed to fully evaluate the association of AR modulation with response to SPT.

### 7.6. Transcriptional Reprogramming and Differentiation

As decreased AR signaling following ligand depletion leads to oncogenic changes in the AR-cistrome, transitioning back from low- to high-T conditions may reprogram the AR-cistrome toward a more differentiated state [97,100]. In addition to showing that AR may directly repress its own expression, the work of Cai et al. described above also showed that agonist liganded AR suppressed the expression of multiple genes mediating DNA synthesis and cell cycle progression, while it increased the expression of genes mediating synthesis of lipids, amino acids, and other metabolic processes. This lead the authors to postulate a model whereby androgen levels in CRPC cells are adequate to stimulate AR activity on enhancer elements of genes mediating certain critical metabolic functions such as lipid synthesis (that are sensitive to lower levels of androgens), but are not adequate to effectively recruit AR and LSD1 to suppressor elements in multiple genes that negatively regulate AR signaling and cellular proliferation. In a subsequent publication this group demonstrated that AR causes transcriptional repression of multiple genes involved in DNA replication via interactions with RB1. In particular, SPT was able to induce AR binding to regulatory regions of genes involved in DNA replication and repress their transcription through recruitment of hypo-phosphorylated RB [101].

Alternatively, Ruan et al. have recently demonstrated that SKP2 can bind and stabilize expression of Twist protein, leading to acquisition of epithelial-mesenchymal transition (EMT) and cancer stem cell (CSC) characteristics [102]. Genetic or pharmacologic depletion of SKP2 reverted these effects and re-sensitized CRPC cells to chemotherapy. Whether AR-mediated repression of MYC, or AR-mediated changes in RB induction of E2F are the upstream factors responsible for the decreased SKP2 expression, these data provide another potential mechanism for the cellular reprogramming of CRPC cells treated with SPT toward a less aggressive tumor biology.

The repression or induction of specific genes involved in transcriptional programming and differentiation has also been linked to androgen induced proliferative arrest in PCa cells. SOX2 is an androgen repressed gene that is upregulated in CRPC and has been shown to stimulate epithelial-to-mesenchymal transition (EMT) as well as mediate lineage plasticity from (AR)-dependent luminal epithelial cells to AR-independent basal-like cells [103,104,105]. Endogenous expression of SOX2 in prostate epithelial cells, human embryonic stem cells, and PCa cells is repressed by AR signaling (via an enhancer element within the SOX promoter), and loss of SOX2 expression has been shown to inhibit growth of the castration-resistant CWR-R1 PCa cell line [103]. While not specifically evaluated, these data suggest a possible role for androgen induced repression of SOX2 in repressing tumor growth via promoting a more differentiated luminal epithelial cell state.

PDS5B (APRIN, AS3) was initially discovered as a gene induced in LNCaP PCa cell lines and rat prostate cells undergoing androgen induced proliferative arrest [5,106]. It was later shown that the basic features of PDS5B (lineage, domain architecture, unique high mobility group (HMG) domains and heterochromatin localization) were consistent with that of a chromatin regulator, suggesting PDS5B may serve as a regulator of chromatin architecture in hormonal differentiation [107]. Although not assessed in PCa models to date, PDS5B was shown to be a critical mediator of differentiation in embryonal carcinoma stem cells, with PDS5B silencing resulting in arrested differentiation at a transient, proliferative progenitor phase characterized by loss of contact signaling, hormone resistance, and continued proliferation [108].

Promyelocytic leukemia zinc finger protein (PLZF, ZBTB16) is another androgen induced transcription factor gene widely involved in regulation of proliferation, differentiation, and stem cell maintenance that could potentially play a role in androgen-mediated growth repression [109]. PLZF expression is rapidly induced by androgen in PCa cell lines, and expression of PLZF has been shown to inhibit proliferation in LNCaP and 22RV1 cells [110,111]. Notably, transcript expression of PDS5B ranges nearly an order of magnitude in CRPC tumors [112], while PLZF expression is reduced/lost in up to 86% of metastatic PCa specimens, with 5–7% of CRPC specimens harboring homozygous PLZF deletions [110,113], suggesting a possible role for androgen-induced expression of PDS5B or PLZF in promoting induction of a more differentiated cell state.

### 7.7. Induction of Cellular Senescence or Quiescence

Senescence is an irreversible cell cycle arrest associated with changes in cell morphology and gene expression that occurs during the normal embryogenesis [114]. Exogenous re-activation and induction of cellular senescence has been proposed as a potential target for cancer therapy [115].

Several mechanisms of induction of senescence by high dose androgens in PCa cells have been described. Roediger et al. demonstrated a dose-dependent induction of G1/G0 cell cycle arrest and senescence associated beta galactosidase activity (SA beta-Gal) in LNCaP, C4-2 and AR-expressing PC-3 PCa cell lines treated with R1881 at 1 nM or higher concentrations, and in malignant prostate tissues treated ex vivo with R1881 at 10 nM or higher concentrations [116]. Formation of senescence-associated heterochromatic foci (SAHFs) has been shown to coincide with stable repression of E2F target genes in a RB-dependent manner, and E2F1 regulates expression of its own gene by a positive feedback loop [117]. Accordingly, they showed E2F1 was localized to SAHF, and further that the p16-RB-E2F1 pathway was required for this effect, as knockdown of p16 by siRNA decreased formation of heterochromtic foci. SA beta-Gal activity was induced after only 3 h of androgen treatment suggesting a non-genomic rapid signaling response, mediated in part via the Rous sarcoma oncogene (SRC)- phosphatidylinositol 3-kinase (PI3K)- serine/threonine kinase 1 (AKT) signaling pathway, as treatment with a SRC, PI3K or AKT inhibitor each abrogated the effect (whereas inhibitors of other factors downstream of SRC e.g., mitogen-activated protein kinase kinase (MEK), mitogen-activated protein kinase 14 (MAPK14, also known as p38), signal transducer and activator of transcription 3 (STAT3), and mechanistic target of rapamycin kinase (mTOR), did not). Mirochnik et al. demonstrated induction of senescence in LNCaP cells and PC-3 cells engineered to express AR via two mechanisms, first, via AR induced expression of p21cip leading, via an unidentifed mechanism, to decreased expression of p63 (a p53-related protein that opposes cellular senescence) and second, via AR induced expression of reactive oxygen species (ROS) leading to decreased RB phosphorylation and repression of E2F target genes [118].

In contrast to senescence, quiescence is a reversible growth arrest in G0/G1 that generally requires persistence of an external growth condition for its maintenance. However, Bui et al. recently reported the androgen-mediated induction of a self-sustained quiescent state in LNCaP and VCaP cells that was dependent on culturing cells at low density, and associated with induction of oxidative stress, a sustained redox imbalance, and transforming growth factor-beta (TGF beta)/bone morphogenic protein (BMP) signaling [119]. Treatment with R1881 induced expression of stress, differentiation and mothers against decapentaplegic homolog (SMAD) signaling markers comprising a dormancy signature that the authors had previously identified in PCa cells rendered quiescent by culture at low density in hypertonic medium. Notably, transient treatment with R1881 at doses greater than 0.2 nM for 7 days caused a sustained decrease in cloning efficiency that persisted for at least 10 days after androgen withdrawal. However, growth arrest could be reversed by treatment with the anti-oxidants glutathione, or *N*-acetylcysteine, or by inhibition of TGF beta/BMP mediated SMAD phosphorylation. The authors propose that utilization of high dose androgen therapy as early as after radical prostatectomy, or possibly biochemical relapse, when cancer cells are still dispersed and solitary may have most efficacy as stable induction of the self-sustained quiescent state in their studies only occurred at low cell density.

### 7.8. Induction of DNA Damage

Although it is well established that ADT causes DNA damage, recent data have also shown that high androgen concentrations induce dsDNA breaks (DSB) in PCa cells that can lead to chromosomal rearrangements such as the TMPRSS2-ERG fusion [120], and may represent one mechanism whereby SPT inhibits proliferation. In particular, studies have shown that androgen signaling leads to co-recruitment of AR and topoisomerase II beta (TOP2B), and to TOP2B-mediated DNA DSBs at regulatory regions of AR target genes in PCa cells [120]. Moreover, treatment of PCa cells with etoposide, a TOP2-inhibitor that prevents resolution of TOP2B-induced DSBs, led to enhanced androgen-induced DSBs in the treated cells [120]. This provided rationale for inclusion of etoposide in the first study of BAT reported in men with CRPC [9]. Although the specific effect of this agent on the response to BAT was not fully dissected, a recent case report documented an extreme response following BAT in a patient with inactivating ataxia telangiectasia mutated (ATM) and breast cancer 2 (BRCA2) mutations, providing support for the concept that DNA damage can sensitize to SPT [121]. These data provide rationale for further studies to test the hypothesis that combining SPT and with DNA damaging agents such as PARP inhibitors (PARPi) will result in clinical responses in men with CRPC.

## 8. High Dose Estrogen Therapy for Breast Cancer–Clinical and Experimental Evidence

Similar to the dual growth-promoting and growth-repressing effects of androgens in PCa, estrogens occupy a similarly paradoxical role in the biology and treatment of breast cancer, although with a more substantial history of clinical use. The efficacy of synthetic estrogens for advanced breast cancer was first described by Haddow in 1944, followed by a number of clinical trials that made estrogens the standard of care in postmenopausal patients with advanced breast cancer from the early 1960s onwards (reviewed in [122,123]). A critical observation in these early studies was the necessity of a ‘gap period’ following development of menopause, in that response rates to diethylstilbestrol (DES, 5–15 mg/day) or ethinyl estradiol (EE, 1.5–3 mg/day) were substantively higher (~30–40%) in women who were at least 5 years post-menopause compared to those who were not (~5–10%).

When the non-steroidal anti-estrogen tamoxifen was introduced in the 1970s for the treatment of advanced breast cancer, it was compared in clinical trials to DES or EE as the current standard of care. Notably, the response rate was generally comparable to that seen with estrogen treatment, but the consistently superior side effect profile of tamoxifen resulted in its uniform adoption for the first line treatment of advanced breast cancer. Estrogen therapy was essentially abandoned until studies in the 1990’s demonstrating the efficacy of this approach in patients who were resistant to anti-estrogens began to emerge. A number of studies evaluating DES or EE in heavily pre-treated post-menopausal women who were resistant to prior hormonal therapies including tamoxifen and/or aromatase inhibitors demonstrated objective response rates in approximately 30% of patients [122,123]. These observations renewed interest in the clinical and biological mechanisms underlying the activity of high dose estrogens in breast cancer.

Similar to the data for PCa, work by several investigators demonstrated that long term adaptation of breast cancer cells in vitro *or* in vivo to estradiol deprivation (and subsequently, to tamoxifen treatment) induces sensitivity to estradiol-mediated growth inhibition [124,125]. In contrast to the relative diversity of mechanisms proposed for androgen-mediated inhibition of PCa cell growth, the primary cause of estrogen-mediated growth inhibition in breast cancer cells appears to apoptosis, albeit via various effector mechanisms [126]. Notably, the conformation of the ER complex, which is dependent on the shape of the estrogenic ligand, can modulate the apoptotic effect, with class I planar estrogens (e.g., estradiol) triggering apoptosis after 24 h and class II angular estrogens (e.g., bisphenol triphenylethylene) delay the process until after 72 h [127].

A number of mechanisms for estrogen-induced apoptosis have been described. Work by the group of C. Jordan has delineated a SRC-dependent estradiol-mediated induction of endoplasmic reticulum stress and inflammatory responses that initiates an unfolded protein response, followed by apoptosis through the intrinsic (mitochondrial) pathway with subsequent recruitment of the extrinsic (death receptor) pathway to complete the process [122]. An estradiol mediated activation of protein kinase AMP-activated catalytic subunit alpha 1 (AMPK) in long term estrogen deprived (LTED) MCF-7 cells has also been described, with increased activity of forkhead box protein O3 (FOXO3) and upregulation of three FOXO3 target genes, Bcl-2-like protein 11 (BIM), fas cell surface death receptor (FAS) ligand (FASL), and Gadd45a (BIM and FASL mediate intrinsic and extrinsic apoptosis respectively and Gadd45a causes cell cycle arrest at the G2/M phase) [128]. Other signaling pathways identified in the apoptotic response to estradiol include estradiol mediated inhibition of PI3K/AKT signaling, nuclear factor kappa b subunit 1 (NF-κB) signaling and the c-Jun N-terminal kinase (JNK) pathway [126].

## 9. Potential Predictive Markers of Response to Androgen Therapy

### 9.1. Androgen Receptor

High AR levels appear to sensitize a number of PC preclinical models to the inhibitory effect of SPT, raising the possibility that tumor AR expression might be useful in predicting response to SPT. Consistent with this hypothesis, high baseline PSA levels were associated with response in the BAT CRPC study, suggesting that SPT responders may have more active AR-signaling [9]. However, as discussed above, not all high AR CRPC models are inhibited by SPT in preclinical studies. The ARCaP cell line expresses low levels of AR yet shows a strong androgen-dependent growth suppression [62], while, studies in AR-expressing PC-3 cells have shown that a growth-inhibitory effect was observed in clonal lines expressing low, moderate and high levels of AR [73], indicating that growth inhibition was not necessarily related to the level of AR overexpression. Furthermore, studies in DU145, a castration resistant PCa cell line that lacks AR, have shown that ectopic overexpression of AR in this cell line failed to yield a growth response to androgen [129].

These data indicate that AR expression by itself is not necessarily correlated with androgen-driven growth suppression, and that a cell must possess the appropriate molecular mechanisms to engage AR as a growth suppressor or as an oncogene [69]. As discussed above, a clear association between response to BAT and modulation of AR-FL or AR-V7 transcript levels was not observed [11], suggesting AR-FL or AR-V7 levels are not a biomarker of SPT response, and the predictive value of AR signaling has yet to be fully evaluated.

### 9.2. DNA Damage Response Genes

As discussed above, preclinical data suggest the induction of dsDNA breaks may be a mechanism mediating the anti-tumor effects of BAT, and an extreme response to BAT in a patient with inactivating mutations in the DNA damage response (DDR) genes ATM and BRCA2 has been reported [121]. However, 50% of men on the BAT studies showed response and it is unlikely all of these had DDR deficiently. Thus the extent to which DDR deficiency predicts for response to SPT-based therapy remains unknown, but may identify a population of patients likely to show the strongest response as well as those most likely to benefit from the combination of SPT with PARP inhibition.

### 9.3. Steroid Metabolism and Transport Genes

Earlier trials using physiologic dose T showed limited clinical activity compared to the supraphysiologic levels achieved in the BAT studies, suggesting the anti-tumor efficacy of SPT may reflect the level of intratumoral androgens achieved on therapy, an effect that may be influenced by steroid transport and metabolizing enzymes such as SLCO1B3 and UGT2B17. Expression and/or genetic variation in SLCO1B3 can modulate cellular T uptake in PCa cells in vitro, and genetic variants of SLCO1B3 linked to more efficient T uptake were associated with a shorter time to progression in men with CRPC on ADT [130,131]. In context of SPT, more efficient tumoral T uptake might associate with an enhanced therapeutic response.

UGT2B17, responsible for the irreversible glucuronidation and ensuing elimination of T and DHT, is highly polymorphic and deletion variants of this enzyme (primarily expressed in the liver) are known to influence circulating steroid levels [132]. As such, patients with deletion variants might sustain higher serum androgen levels following exogenous T dosing. UGT2B17 is also expressed in primary and CRPC tumors, where its in situ tumor activity could also influence the maintenance of tumor androgen levels [133,134]. Thus, genetic variation in genes such as SLCO1B3 or UGT2B17 may serve as predictors of response to SPT.

## 10. Future Directions

Although a significant body of preclinical evidence, and emerging clinical data, support the concept of high dose androgen therapy for the treatment of CRPC, a number of important questions remain unanswered. What is the optimal dosing schedule of T therapy? Given the diversity of mechanisms observed in preclinical studies, what are the specific mechanisms mediating clinical anti-tumor efficacy in a particular individual? Can rational drug combinations be designed to improve efficacy or sensitize tumors that are not inhibited by high dose androgen alone? Can biomarkers be identified to predict response or resistance to SPT? What, if any, is the impact of stromal AR signaling on the response to high dose androgen therapy?

The relative success of the modern studies employing the BAT approach are encouraging, but the extent to which the clinical benefit observed in these studies reflects the bipolar dosing strategy or the documented achievement of truly supra-physiological androgen levels remains unclear. Preclinical studies have consistently found that SPT delivered on a continuous basis represses the growth of PCa cells, and no studies (preclinical or clinical) have directly compared continuously administered SPT with BAT. Whether rapid cycling of SPT (vs. continuous SPT) is necessary to achieve the clinical benefit or delays resistance by preventing adaptive AR-downregulation remains to be determined. Ultimately, the predominant mechanism of SPT action may dictate the ideal dosing strategy, with continuous treatment likely providing improved efficacy if the main mechanism of SPT action relates to changing from a “low-T” oncogenic AR transcriptome to that of a more differentiating SPT transcriptome [97], and BAT demonstrating more activity if repeated cycles of DNA damage is a critical mechanism of action. However, continuous SPT can repress the expression of genes that repair DNA damage, and thus the continuous repression of these repair programs may also exceed the responses seen with BAT.

Understanding the mechanisms driving the anti-tumor efficacy of high dose androgen is particularly relevant for designing rational drug combinations. For example, the observation that androgens can induce DSB in conjunction with a recent report of an extreme response to BAT in a patient with inactivating ATM and BRCA2 mutations, provides support for the concept that DNA damage can sensitize to SPT [121]. These data provide a clear rationale for combining SPT with DNA damaging agents such as PARP inhibitors (PARPi) and such a trial is currently being designed. However, rational combinations based on other proposed mechanisms can also be conceived, including combinations with cell cycle inhibitors to promote the impact of high dose androgen in mediating cell cycle arrest, combinations with MYC inhibitors to promote the MYC-repressing effects of high dose androgens, or combinations with proteasome inhibitors to prevent AR degradation and promote the proposed stabilization of AR on DNA and inhibition of DNA licensing induced by high dose androgens.

A further consideration which merits discussion is the potential for aromatase-mediated conversion of exogenous T to E2. PCa cells can variably express one or both of ER-alpha (which can promote PCa proliferation) and ER-beta (which can inhibit PCa proliferation); thus the net effect of a possible increase in estrogen signaling may be adverse or beneficial depending on the relative level of each [66]. Whether this is a clinically relevant concern is unclear, as the extent to which T undergoes intra-tumoral conversion to E2 in men treated with SPT is unknown. However, studies testing the combination of T with an aromatase inhibitor (either upfront or at evidence of disease progression) would be informative.

The potential ability of T therapy to re-sensitize CRPC tumors to AR-axis inhibition is intriguing, and if borne out, may represent an important clinical approach for delaying disease progression. Illustrating the potential ability of androgen-repletion to ‘ug’ mechanisms of androgen sensitivity, culture of the androgen-repressed LNCaP 104-R1 cells in androgen rich media gave rise to a subline that was again androgen-sensitive for growth [80]. (Importantly, the related androgen-repressed LNCaP 104-R2 line passaged under similar androgen rich conditions gave rise to a subline that was androgen-insensitive for growth or repression, demonstrating that androgen induced effects on androgen sensitivity are not uniform.) In clinical studies a high response to AR-signaling inhibition was seen in men after BAT therapy [9,11] although whether this reflects a change to a more differentiated phenotype or specific changes in the AR/co-regulator signaling apparatus resulting in re-establishment of androgen-sensitivity remains to be determined.

Finally, preclinical studies of androgen-mediated PCa repression have largely been carried out in vitro or in subcutaneous xenograft models, systems which do not take into account the role of in situ stromal AR signaling on PCa behavior [135]. Notably, multiple studies have found that lower AR expression in PCa stroma is associated with disease progression and/or worse outcome, implying that stromal AR is protective [136]. Mechanistically, in the absence of stromal AR signaling, the fibroblast-derived extra-cellular matrix (ECM) was shown to have a decreased capacity to promote attachment of both myofibroblasts and cancer cells, and was less likely to impede cancer cell invasion [137]. In a separate study, AR-depleted cancer associated fibroblasts (CAFs) promoted increased stem cell marker expression in human PCa cells, apparently via increased levels of Interferon gamma (IFN-γ) and macrophage colony-stimulating factor (M-CSF) [138]. Thus, to the extent AR signaling in PCa stroma maintains an ECM microenvironment inhibitory to cancer cell invasion, or restrains induction of stem cell characteristics, enhanced AR-mediated stromal signaling may also contribute to the anti-tumor activity of high dose androgen therapy observed in clinical studies. To date these hypotheses remain unexplored.

## 11. Conclusions

Meaningful clinical responses have now been observed in men with PCa treated with high dose T, but studies designed to determine the molecular mechanism(s) driving these responses and identify predictive biomarkers are needed in order to optimize this approach and identify rational treatment combinations.

The clinical utility of potential treatment combinations will clearly depend on the relative importance of the various proposed mechanism, and the extent to which the diversity of mechanisms observed in the laboratory is recapitulated in human tumor specimens. Clinical studies with built-in collection of biospecimens will be critical to assessing the molecular changes associated with response or resistance to SPT, and for identifying potential predictors of response. There may be subsets of men whose tumors will be variably responsive to SPT or particular combinations based on the status of steroid transport and metabolizing genes, DNA damage repair genes, AR expression, MYC dependence, and/or RB loss or alterations in other cell cycle regulators. Ultimately, high dose T therapy is likely to represent yet another avenue for applying the principles of precision medicine for optimizing the care of men with CRPC.

## Figures and Tables

**Table 1:** Contemporary Trials of High Dose Androgen Therapy.

Patient Population	No. of Patients	Treatment Regimen	Serum T Level	PSA Response	Objective Response	Median Time to Progression	Caner Related Adverse Effects	Ref.
CRPC (disease burden or symptoms not designated)	12	T via 5 mg transdermal patch or 1% gel for 1 week, 1 month, or until disease progression	physiologic (342–876 ng/dL)	1 patient had PSA decline >50% from baseline	none	84 days (23–247 days)		[65]
CRPC with minimal metastatic disease	15	transdermal T at 25, 5.0 or 75 mg/day	physiologic (94–824 ng/dL)	3/15 (20%) had PSA declines from baseline (largest decline 43%)	none	63 days (14–672 days)	one patient with symptomatic progression	[66]
Asymptomatic CRPC with low to moderate metastatic burden	16	T (400 mg IM day 1 of 28) and etoposide (100 mg oral daily; days 1 to 14 of 28)	T > 1500 ng/dL (~50 nM) at 2 days after T injection (range 920 to >3200 ng/dL), above 600 ng/dL at 2 weeks, and 150 ng/dL by 28 days	7/14 (50%) had PSA declines from baseline (≥50%)	radiographic responses in 5/10 (50%), and 4 continued on treatment for ≥1 year	11 months (3 to not reached)	2 patients were not evaluable because they came off study after only one cycle of therapy due to toxicity	[9]
CRPC post progression on enzalutamide	30	alternating 3 month cycles of BAT (T 400 mg IM on days 1, 29 or 57), followed by 3 months of ADT alone	not reported	9/30 (30%) men achieved a ≥50% decline in PSA from baseline	50% of patients achieving an objective radiographic response	8.6 months (4.7 to not reached)	3 patients progressed per RECIST criteria and 3 had unconfirmed progression on bone scan	[11]
Asymptomatic hormone naïve with low metastatic burden or biochemically recurrent disease, who achieved PSA <4 ng/dL after 6 months of ADT	29	T 400 mg IM on days 1, 29, and 57	not reported	17/29 (59%) achieved primary endpoint of PSA < 4 ng/dL after 18 months	4 of 10 evaluable patients had complete and 4 had partial responses (80%)	not given	3 patients taken off study prior to completing 2 cycles due to concerns for early progression	[10]

CRPC: castration resistant prostate cancer; T: testosterone; PSA: prostate specific antigen; BAT: bipolar androgen therapy; IM: intra-muscular; ADT: androgen deprivation therapy.

## Appendix A

### Appendix A.1. Preclinical Observations on Androgen-Mediated Growth Repression of Prostate Cancer

Preclinical studies demonstrating the repressive effect of androgen on prostate cancer growth are summarized in the primary text of the manuscript. For the interested reader, and to more fully illustrate the similarities and heterogeneity of response observed, a more detailed review of findings in each pre-clinical model is provided in this Appendix A and summarized in Appendix Table A1.

#### Appendix A.1.1. LNCaPs

Derived from a supraclavicular lymph node metastasis in a 50-year-old Caucasian male [139], LNCaP is the most widely used cell line in prostate cancer research. A number of sublines have been derived under different culture conditions and with varying degrees of sensitivity to androgen induced growth and repression. Horoszewicz et al. originally reported the stimulatory effect of DHT on LNCaPs in androgen-depleted conditions 5% charcoal stripped serum (CSS) and the dose-dependent suppression in response to DHT in cells cultured in relatively androgen-rich conditions (5% FBS) [140]. De Launoit et al. also demonstrated a progressive decrease in the stimulatory effect of DHT (in 2% CSS) at doses higher than 0.1 nM, returning to basal levels with doses between 1 nM and 100 nM [78].

##### I. LNCaP 104-S, 104-R1, 104-R2, R1Ad, R2Ad

Similarly, androgen sensitive LNCaP 104-S cells showed maximal proliferation at 0.1 nM R1881 (with progressive growth suppression with higher doses), while 104-R1 and 104-R2 cells (derived after passages in androgen-depleted medium for 10 months and 18 months, respectively) proliferated more rapidly than 104-S cells in androgen-free conditions, and were severely inhibited by 0.1 nM or higher R1881 doses [57,58], illustrating the left-shift in androgen-repressed sensitivity frequently observed in the transition from androgen-sensitive to androgen-insensitive cell growth. Consistent with the in vitro data, proliferation of LNCaP 104-S tumors in vivo was stimulated by androgens but testosterone propionate (TP) pellets implanted in castrated nude mice bearing LNCaP 104-R2 resulted in tumor growth inhibition and a significantly reduced tumor size [7].

Interestingly, when re-passaged in androgen-rich conditions (castrated mice bearing T pellets), 104-R1 cells (now called R1Ad) lost the androgen-repressed phenotype and showed androgen-induced growth in vivo, illustrating the potential ability of androgen-repletion to ‘re-engage’ mechanisms of androgen sensitivity. In contrast, however, 104-R2 cells passaged in androgen-rich conditions gave rise to the androgen-insensitive R2Ad subline, the growth of which was not affected by R1881 or the anti-androgen bicalutamide [80], demonstrating that androgen-induced effects on androgen sensitivity are far from uniform.

**Table A1:** Preclinical Responses to Androgen-Mediated Growth Repression.

Cell Line	Source	Derivation	In Vitro Growth Characteristics	In Vivo Growth Characteristics	Refs.
LNCaP	Lymph node metastasis in a 50-year-old Caucasian male with CRPC		Biphasic response in CSS (peak stimulation at 0.1 nM DHT, progressive growth suppression at 1 nM to 100 nM). Androgen repressed in 5% FBS		[78,139,140]
104-S	LNCaP	Parental Line	Similar to original report Biphasic response in CSS (peak stimulation at 0.1 nM R1881, growth suppression at higher doses)	In vivo growth stimulated by androgens	[7,58]
104-R1	LNCaP 104-S	Passage in CSS × 10 mo	Proliferated more rapidly than 104-S cells in CSS Severely growth repressed by 0.1 nM or higher R1881 doses	In vivo growth inhibited by androgens	[7,57,58]
104-R2	LNCaP 104-S	Passage in CSS × 18 mo			
R1Ad	LNCaP 104-R1	Re-growth in castrate mice after T treatment in vivo	Lost androgen-repressed phenotype Androgen sensitive for growth		[141]
R2Ad	LNCaP 104-R2	Re-growth in castrate mice after T treatment in vivo	Lost androgen-repressed phenotype Androgen insensitive for growth-not affected by R1881 or bicalutamide		[80]
MOP	LNCaP	Passage (of LNCaP passage 25 cells) in CSS × 10–12 mo	Androgen insensitive for growth Dose-dependent growth suppression in response to R1881 at 0.1 to 10 nM	In vivo growth inhibited by androgens	[75]
JAC	LNCaP	Passage (of LNCaP passage 55 cells) in CSS × 10–12 mo			[76]
ME	MOP	Regrowth in castrate mice after T treatment in vivo	Still showed androgen repressed growth in vitro		[76]
LNCaP-abl	LNCaP	Long term passage in CSS	Biphasic response but with higher sensitivity than parental LNCaP (max proliferation at 0.001 nM R1881 vs. 0.01 nM)		[142]
CWR22	Primary PCa tumors initially injected subcutaneously into nude mice supplemented with T, then serially transplanted as cell suspension		Biphasic response to androgen, with optimal proliferation at 25 to 35 nM testosterone and growth repression at concentrations higher than 35 nM		[61,143,144]
CWR22R	CWR22	Derived from a CWR22 tumor showing castration resistant re-growth in vivo	Not consistently stimulated by androgen Growth repressive effect left-shifted vs. parental CWR22 line, with repression induced at T levels of approximately 25 nM		[145]
22RV1	CWR22R		Androgen-sensitive for growth without a biphasic response		[63]
ARCaP (MDA PCa 1)	Isolated from the ascites fluid of an 83-year-old Caucasian man with metastatic CRPC		Highly androgen-repressed growth (starting as low as 100 pM DHT) despite relatively low AR expression	Grew 3 times faster in castrated hosts than in intact male hosts; growth in castrated hosts was suppressed by exogenous T	[62]
VCaP	From a vertebral metastatic lesion of patient with CRPC		40% repression at 10 nM R1881. Detachment and disintegration of cells passaged in low androgen conditions (10% FBS) when treated with 1 nM T in vitro	Poor growth in intact (noncastrate) SCID mice [ 56]	[56,67,146]
E006AA	From primary tumor of a 50-year-old African-American man with clinically localized PCa		Biphasic response, with proliferative response as low as 1 fM DHT and maximal proliferative at 0.1 pM DHT		[147]
MDA PCa 2b	From a bone metastasis of a patient with CRPC		Biphasic response, peak proliferation at 10 nM DHT with growth inhibitory effects at higher concentrations	Stopped growing or decreased in size after castration (response to high dose androgen not evaluated in vivo)	[59,60]
MDA PCa 2b-hr	MDA PCa 2b	culture of MDA PCa 2b in CSS for 35 weeks	Biphasic response to T concentrations ranging from 0.1 ng/ml to 1000 ng/ml, with maximal proliferation 1 ng/mL T		[60]
RC-77T	From primary tumor of a 63-year-old African American man with clinically localized PCa		Biphasic response, maximal growth at 0.1 nM R1881 and growth inhibition at higher doses		[148]
PC3-AR	From lumbar vertebral metastasis of a 62-year-old white man	PC3 with exogenous expression of AR	Androgen mediated growth repression at DHT 0.1 nM	In vivo growth inhibited by androgen levels presesnt in intact male mice	[129,149,150,151]

CSS: charcoal stripped serum; DHT: dihydrotestosterone; FBS: fetal bovine serum; AR: androgen receptor; SCID: severe combined immunodeficiency; PCa: prostate cancer; MDA: MD Anderson.

##### II. MOP, JAC, ME

LNCaP variant MOP and JAC cells (derived, respectively, by continuous passaging of androgen-sensitive LNCaPs at passage 28 and 55 in 2.5–5% CSS for 10–12 months), were androgen-insensitive for growth, and showed dose-dependent growth suppression in response to R1881 at 0.1 to 10 nM [75,76] Similarly, testosterone treatment delayed the take of palpable tumors following injection of LNCaP variant MOP cells in female nu/nu mice in vivo. Notably, tumors eventually escaped treatment, but ME cell lines, established from these non-testosterone repressed tumors still showed androgen repressed growth in vitro [76].

##### III. LNCaP-abl

Similar to androgen-sensitive LNCaP cells, LNCaP-abl, another subline derived from long-term passage in CSS, demonstrated a biphasic response to androgen, but with a higher sensitivity than the parental LNCaP [142], again illustrating the left-shift in androgen-repressed sensitivity observed in the transition from androgen-sensitive to androgen-insensitive cell growth. While the maximum proliferative rate of the parental LNCaP was achieved at 0.01 nM R1881, the R1881 dose that achieved maximal proliferation of the abl subline was left-shifted one order of magnitude to be at 0.001 nM. Moreover, at passages higher than 75, androgen treatment only induced an inhibitory growth effect on the LNCaP-abl subline [142].

##### IV. C4-2B

The C4-2B cell line was isolated from a mouse vertebral metastasis in 1994 as a subline of a LNCaP derivative established by Wu et al. [151]. Wu et al. co-injected LNCaP and osteosarcoma cell lines subcutaneously in intact mice, followed by castration, resulting in growth of an androgen-independent tumor. Cells cultured from these tumors, denoted C4 cells, were then subcutaneously injected in castrate mice, giving rise to tumors from which the C4-2 cells were cultured. Following subcutaneous or orthotopic injection of C4-2 cells into castrated mice, cells isolated from a vertebral bone metastasis were cultured and denoted C4-2B [152]. Although Thalmann et al. reported that the osseous metastases of the androgen-independent C4-2 cells (C4-2B) were enhanced in castrated hosts, suggesting androgen suppression of androgen-independent dissemination [152], Pfitzenmaier et al. later reported that the growth of C4-2 metastases was inhibited by androgen suppression i.e., castration [153].

#### Appendix A.1.2. CWR22 and CWR22R

CWR22 is a serially transplantable xenograft established along with other CWR lines from primary tumors excised through transurethral resection of the prostate or radical prostatectomies, and [143,144]. However, it has been since transplanted through injection of the cell suspension into testosterone supplemented nude mice [144]. After castration, the xenograft markedly regresses, but is often followed by tumor relapse 3 to 10 months after castration [61,145], from which the CWR22R line was derived.

Nagabhushan et al. reported the differential sensitivity of CWR22 and CWR22R cells to androgen stimulation in soft agar [61], again illustrating the left-shift in androgen-repressed sensitivity observed in the transition from androgen-sensitive to androgen-insensitive cell growth. CWR22 cells showed a biphasic response to androgen, with optimal proliferation at 25 to 35 nM testosterone and growth repression at concentrations higher than 35 nM [61]. Growth of CWR22 in FBS was parallel to CSS curves, with more overall cell proliferation in FBS. In both media, T concentrations higher than 35 nM inhibited proliferation [61]. In contrast, CWR22R cells were not consistently stimulated by androgen and the growth repressive effect was left-shifted compared to the parental CWR22 line, with repression induced at T levels of approximately 25 nM [61]. In contradistinction to CWR22 cells, 22Rv1, another AI line derived from CWR22R, is androgen sensitive for growth without a biphasic response [63].

#### Appendix A.1.3. ARCaP

Isolated from the ascites fluid of an 83-year-old Caucasian man with metastatic PCa, the highly metastatic ARCaP cell line, also known as MDA PCa 1, was first introduced and characterized by Zhau et al. in 1996 [62] and is notable for its highly androgen-repressed phenotype despite relatively low AR expression. ARCaP cells have been reported to demonstrate growth repression to DHT in vitro in a concentration-dependent manner (starting as low as 100 pM) [62]. Interestingly, overexpression of AR in these cells restored a biphasic response to androgen, with stimulation of proliferation in response to R1881 at 0.1 nM to 10 nM but suppression of proliferation at higher R1881 concentrations of 100 nM to 1 uM R1881 [72]. ARCaP tumors, when maintained as subcutaneous xenografts, grew 3 times faster in castrated hosts than in intact male hosts, suggesting the sensitivity of these cells to suppression by physiological levels of androgen. Consistent with the in vitro assays, tumor growth in castrated hosts was suppressed by subcutaneous administration of either testosterone propionate [62].

#### Appendix A.1.4. VCaP

These cells were isolated in 2001 from a vertebral metastatic lesion of a patient with CRPC. Initial studies characterizing this line demonstrated it was androgen sensitive for growth in vitro and in vivo but did not report a biphasic response [146]. Subsequent studies by several groups have demonstrated the ability of high dose androgen to repress growth in this model. Denmeade showed 40% repression of growth after 5 days of exposure to 10 nM R1881 [67]. Thelen et al. demonstrated detachment and disintegration of VCaP cells passaged in low androgen conditions (10% FBS) when treated with 1 nM T in vitro, and poor growth when injected subcutaneously in intact (noncastrate) severe combined immunodeficiency (SCID) mice [56].

#### Appendix A.1.5. E006AA

This cell line was established by Koochekpour et al. as spontaneously immortalized cells from a 50-year-old African-American patient who underwent radical retro-pubic prostatectomy for treatment of a clinically localized prostate cancer [147]. In vitro studies have shown a biphasic response of E006AA to androgen, with the cells being much more sensitive to DHT stimulation (in 1% CSS) than were LNCaP cells. The proliferative response of E006AA started at concentrations as low as 1 fM DHT, with a maximal proliferative effect at 0.1 pM, while higher DHT concentrations had an inhibitory effect on cell number compared with untreated cells [147].

Koochekpour et al. reported E006AA cells were non-tumorigenic in vivo when xenografted in athymic nude mice [147]. D’Antonio et al. subsequently showed that these cells tumorigenic in the NOD-SCID-IL2Rgamma (NSG)triple deficient mice, attributing the failure of growth in the original studies to the fact that nude mice possess high levels of activated natural killer T-cells, resulting in increased host-immunoreactivity towards a growing tumor [154]. Moreover, and despite having been isolated from a hormonally naïve primary prostate cancer, E006AA cells showed castration-resistant growth when xenografted in castrated vs. intact NSG mice [154]. However, the response of tumors to exogenous androgen in vivo, either in castrated or intact mice, has not been assessed.

#### Appendix A.1.6. MDA PCa 2b and MDA PCa 2b-hr

MDA PCa 2a and 2b were derived from a bone metastasis of a patient with castration resistant prostate cancer. Both lines express AR, grow in vitro and in vivo, and are androgen sensitive [59]. Both lines demonstrate peak proliferation in response to DHT at 10 nM with growth inhibitory effects at higher concentrations [59].

The MDA PCa 2b-hr cell line was derived from the androgen-dependent MDA PCa 2b cell line after prolonged culturing in androgen-depleted media [60]. Hara et al. reported the biphasic response of MDA PCa 2b-hr cells to testosterone concentrations ranging from 0.1 ng/mL to 1000 ng/mL, with the maximal proliferation rate achieved with 1 ng/mL of testosterone [60]. Navone et al. reported that MDA PCa 2b tumors formed in athymic mice stopped growing or decreased in size after castration [59]. Conversely, Hara et al. reported that in nude mice bearing MDA PCa 2b tumors, treatment with a 100 mg DHEA pellet (which achieved a 12.4 ng/mL serum testosterone levels, comparable with the physiological levels in uncastrated men) stimulated MDA PCa 2b growth. However, the effect of exogenous high-dose androgen on tumor growth in castrated conditions has not been assessed in this model.

#### Appendix A.1.7. RC-77N/E & RC-77T/E

These cell lines were first introduced by Theodore et al. in 2010 [155]. Tumor tissue (RC-77T) and non-malignant tissue (RC-77N) used for generating the cell lines that were obtained from a radical prostatectomy specimen of a 63-year-old African American patient with a clinical stage T3c adenocarcinoma with poor differentiation (Gleason 7) [155]. Androgen sensitivity assays were carried out in keratinocyte serum-free media (K-SFM) in the presence of 0, 0.1, and 1 nM R1881. RC-77T cells were shown to be more sensitive to androgen stimulation, reaching a peak growth with 0.1 nM R1881, than were RC-77N cells, which reached their peak growth with 1 nM. Higher doses (10–100 nM R1881) seemed to inhibit cell growth [155]. Effects of exogenous androgens on growth in vivo have not been reported.

#### Appendix A.1.8. PC-3

PC-3 cells were first derived by Kaighn et al. from a lumbar vertebral metastasis in a 62-year-old white man in 1979 [148]. Although the original PC-3 cell line lacks AR, multiple studies have used exogenous AR expression in PC-3 cells to investigate the role of AR in the androgen-mediated growth response of the cells. Early studies reported that ectopic expression of AR using a viral promoter in PC-3 cells led to androgen-mediated suppression of cell growth [73,156,157]. Yuan et al. first reported more than a 50% decrease in proliferation of PC-3 cells transfected with human full length AR when treated with 2 ug /mL (6.8 uM) DHT for 72 h, and more than 40% inhibition of cell proliferation with DHT doses as low as 0.1 nM after a 72 h incubation [156]. These findings were confirmed later by Litvinov et al. using a modified expression vector for the exogenous AR expression [129,149]. Interestingly however, Altuwaijri et al. reported an otherwise slight androgen-induced cell growth of PC-3 cells expressing AR driven by its natural human AR promoter, denoted PC-3(AR)9 cells, when treated with 1 nmol/L DHT [150]. To confirm the AR-mediated growth inhibition in animal models, Litvinov et al. xenografted Lenti-AR PC-3 cells and control cells into male nude intact mice. PC-3-Lenti-AR tumors in mice were profoundly growth inhibited in comparison with PC-3 control tumors [129].
